# Ubiquitin signatures on aggregating proteins in neurodegeneration

**DOI:** 10.1042/EBC20253046

**Published:** 2026-03-16

**Authors:** Subhashree Sahoo, Amrita Arpita Padhy, Kummari Shivani, Ashish Misra, Parul Mishra

**Affiliations:** 1 Department of Animal Biology, School of Life Sciences, University of Hyderabad, Hyderabad, 500046, India; 2 Department of Biotechnology, Indian Institute of Technology Hyderabad, Kandi, Sangareddy, 502285, India

**Keywords:** autophagy, neurodegenerative disease, proteasome, ubiquitin, ubiquitin E3 ligases

## Abstract

The aberrant accumulation of misfolded proteins marked by cellular dysfunction and progressive neuronal loss is the hallmark of neurodegenerative diseases including Alzheimer’s disease, Parkinson’s disease, Huntington’s disease and amyotrophic lateral sclerosis. This review examines the pivotal role of ubiquitin modifications in altering the fate of aggregation-prone proteins such as tau, α-synuclein, mutant huntingtin, TAR DNA-binding protein 43 and superoxide dismutase 1. The ubiquitin signatures identified by their linkage types, chain architectures and site specificities emerge as a complex regulatory language that influences the clearance, aggregation or cellular propagation of these aggregating proteins. The dysregulation of other components of the ubiquitin association pathways, such as impaired E3 ligases and deubiquitinases, also contributes to the inefficient protein disposal and disease progression. Understanding how ubiquitin signatures alter the spatiotemporal dynamics of aggregating proteins is critical for advancing our knowledge of disease biology. Here, we focus on the role of ubiquitin modifications and their associated regulators affecting protein fate and neurotoxicity, and highlight the current therapeutic strategies targeting the degradation of aggregating proteins to uncover potential avenues for treating neurodegenerative diseases.

## Introduction

Neurodegenerative diseases such as Parkinson’s disease (PD), Alzheimer’s disease (AD), Huntington’s disease (HD), amyotrophic lateral sclerosis (ALS) and frontotemporal dementia (FTD) are characterized by the progressive accumulation of misfolded proteins and the decline of cellular proteostasis [1–3]. While chaperones maintain proteins in their folded and functional conformations, ubiquitin signalling is the key regulatory mechanism governing the cellular fate of both folded and misfolded proteins [4–6]. The distinct patterns of ubiquitin association with substrate proteins are referred to as ‘ubiquitin signatures’ or ‘ubiquitin codes’, which describe the ubiquitin topologies that selectively guide the stability, interaction networks, localization and degradation of target proteins during both healthy and disease conditions [6].

Ubiquitin codes regulate essential processes in neurons including synaptic plasticity, axonal transport, mitochondrial dynamics and the functions of key neurogenic transcription factors [7–9]. Dysfunctional ubiquitin signalling has been directly implicated in neurodegeneration. One of the strongest and early pieces of evidence for this was provided by the identification of ubiquitin-positive protein inclusions in the post-mortem brain tissues of patients with neuronal degeneration [10–13]. These inclusions represented ubiquitinated forms of different proteins such as aggregated α-synuclein in Lewy bodies associated with PD, hyper-phosphorylated tau in neurofibrillary tangles in AD, polyQ expanded Huntington’s aggregates in HD, cytoplasmic inclusions of TAR DNA-binding protein 43 (TDP-43) and Fused in sarcoma (FUS) in ALS and FTD [2]. These initial pathological findings strongly suggested that ubiquitination is part of aggregate processing, but whether it is a cause or consequence of disease progression remains largely unknown.

Numerous mutations identified in ubiquitin E3 ligases and deubiquitinases (DuBs) which regulate chain assembly and editing also emphasize on the role of ubiquitin signatures in neurodegeneration [3,14]. These mutant enzymes abundantly accumulate in the neurons which are highly vulnerable to proteotoxic stress due to their long lifespan and post-mitotic nature. It is well established that inhibition of E3 ligases, proteasome and lysosome disrupts effective degradation of toxic protein aggregates, further worsening neurotoxicity [3,15–20]. This suggests that ubiquitin modification can either target aggregates for degradation or contribute to their unnatural persistence depending on the nature of ubiquitin signature and its cellular context. Thus, it is critical to investigate the ubiquitin topologies on each disease-associated protein by using robust analytical tools to learn how these protein aggregates nucleate, grow and persist, leading to neuronal death [21–23]. Understanding the ubiquitin signatures will provide insights into disease progression and highlight potential therapeutic strategies for modulating the ubiquitin associated cellular networks. Recent research has focused on promising therapeutic approaches targeting the ubiquitin signalling. These approaches include small molecules that modulate ubiquitin E3 ligases and DUBs, strategies that bias chain formation toward degradation competent linkages and engineered systems such as proteolysis-targeting chimeras (PROTACs), which recruit ubiquitin ligases to selectively eliminate pathological proteins [2,24,25]

In this review, we provide a comprehensive overview of the ubiquitin signatures on key aggregating proteins implicated in neurodegeneration and discuss how these signatures influence the cellular fate of aggregating proteins, ranging from function to toxicity. We also highlight the emerging therapeutic strategies targeting ubiquitin modifications to alleviate neurodegeneration.

### Ubiquitin signature: A sophisticated language for proteostasis

Ubiquitination has evolved from a passive degradation tag into a sophisticated language for inter and intracellular communication. Ubiquitin associates covalently by its C-terminal tail to the lysine residues of the substrate protein [26]. Emerging evidence shows that ubiquitin can also associate with proteins via their cysteine, serine, threonine or N-terminal methionine, although these non-lysine linkages are not well mapped on classical neurodegenerative aggregates [27]. Ubiquitination involves multiple steps, including ubiquitin activation, conjugation and ligation to substrates mediated by a vast and diverse array of E1, E2 and E3 enzymes respectively (known as code ‘writers’), with the E3 enzymes conferring substrate specificity. Ubiquitin itself contains seven lysine residues (K6, K11, K27, K29, K33, K48, K63) and upon substrate association, it can self-associate via one or many lysine or even its N-terminal methionine (M1), giving rise to homo or hetero-polyubiquitin chains of distinct conformations [28,29]. These chain topologies are known to govern distinct cellular functions of their associated proteins. K48 and K63 polyubiquitinated proteins are targeted for degradation via the proteasome and autophagosome, respectively [30,31]. The K11 ubiquitin chains regulate cell cycle progression and ER-associated degradation (ERAD) [32,33], while the M1-linked (linear) chains play critical roles in immune signalling and NF-κB pathway activation. Monoubiquitin (single ubiquitin on any one lysine of the substrate protein) and multi-monoubiquitin (single ubiquitin on multiple lysines of the substrate protein) patterns are known to regulate membrane trafficking, histone regulation and receptor internalization [34–36]. Interestingly, these diverse ubiquitin signals are carefully interpreted by ubiquitin-binding domains (UBDs) present in the ‘code readers’ which decode the ‘message’ and initiate appropriate downstream responses. The ubiquitin chain architecture is constantly edited by a set of deubiquitinating enzymes (known as ‘eraser’) that selectively release free ubiquitin from the linkages to maintain a delicate balance between the free and conjugated pools of cellular ubiquitin [37–39]. It is this dynamic and reversible nature of the ubiquitin signature that enables the cells to rapidly adjust protein fate decisions in response to environmental cues, thereby maintaining cellular homeostasis. The constant identification of novel mixed-chain topologies and their cellular implications strongly suggests the need for a deeper understanding of ubiquitin-dependent processes in health and disease.

### Ubiquitin signatures for general neuronal functions

Ubiquitination helps maintain various aspects of neuronal health including neuronal development and differentiation, synaptic communication, organelle homeostasis, axonal transport and most importantly, the regulation of toxic protein aggregates. Of all the linkages, K48, K63 and K11 are the most abundant chains identified in rat brain [40]. Proteasomal degradation of K48-linked REST (RE1-Silencing Transcription Factor) in neural progenitor cells enables the expression of neuron-specific genes allowing for neuronal differentiation [41]. Notch preserves the neural progenitor pool by preventing premature differentiation, while Wnt/β-catenin promotes the proliferation of progenitor cells. Proteasomal targeting of Notch intracellular domain (NICD) and β-catenin leads to neurogenesis and differentiation of neural precursor cells. These processes of neuronal fate determination are primarily regulated by K48 and K11-linked ubiquitin chains. K48 linkages also regulate the turnover of synaptic proteins such as neurotransmitter receptors, scaffolding proteins and SNAREs critical for synapse formation and plasticity. Accumulation of these linkages triggers signals for presynaptic clustering and synaptic transmission in neurons [42]. A recent study shows that K63 ubiquitin chains decorate vesicles containing neurotransmitters, allowing for neuronal communication at the synaptic cleft [43]. K63 linkages also promote axonal growth by restricting the localization of an RNA binding protein, muscleblind-like protein 1 (MBNL1), to the cytoplasm where it enhances the translation of axon growth promoting mRNAs [44]. Another aspect of neuronal function is its high metabolic requirements, making mitochondria indispensable for neuronal energy supply. Linear ubiquitin linkages or ubiquitin chains of K6, K11, K27 and K63 topologies are demonstrated to be recruited by Parkin (an E3 ligase)-PINK1 on damaged mitochondria which triggers mitophagy to ensure proper neuronal functions. K63 linkages enabled by NEDD4 and TRAF6 also regulate the endocytosis and signal transduction functions of many neuronal proteins. We have cited a few examples of ubiquitin signatures regulating general neuronal functions, but these aspects of neuronal health regulated by ubiquitination are discussed in detail in other reviews [3,45–48].

### Ubiquitin signatures on aggregating proteins in neurodegeneration

**Figure 1:**
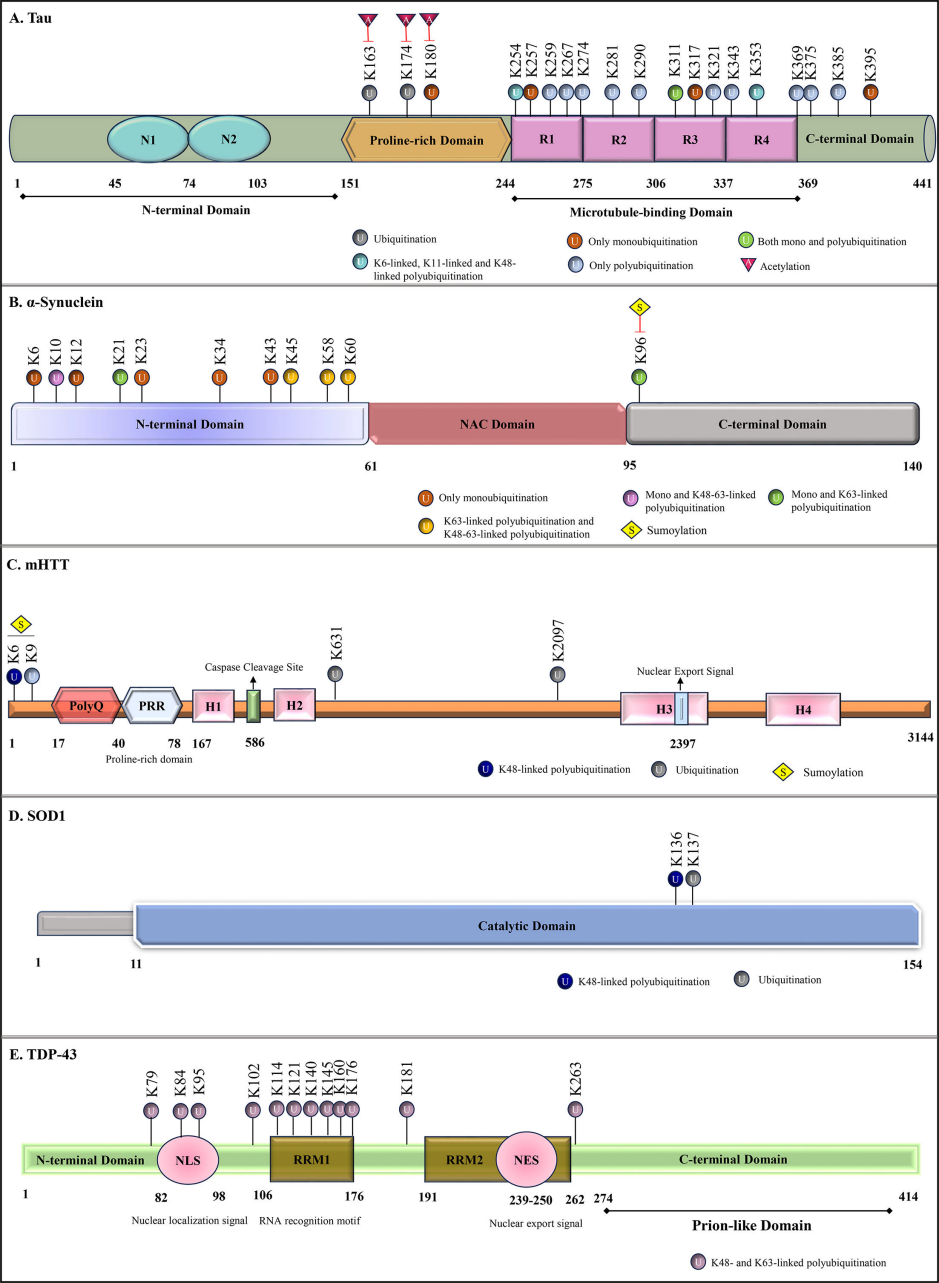
Ubiquitination sites on various aggregating proteins.

Neurons rely on continuous protein synthesis to support their long lifespan, making them vulnerable to translational errors and stress-induced misfolding. The resulting accumulation of toxic aggregates overwhelms the ubiquitin-proteasome system and autophagy pathways, ultimately driving the neuronal loss which is characteristic of neurodegenerative diseases. This review focuses on tau, α-synuclein, mHTT, SOD1 and TDP-43 because they are the core pathogenic proteins underpinning the major neurodegenerative diseases and have the most clearly defined ubiquitin modification patterns. For each of these proteins, specific ubiquitin linkages have been mechanistically linked to their aggregation behaviour, turnover pathways and neuronal toxicity as shown in Figure 1 and Table 1. These proteins effectively exemplify how ubiquitin modifications operate across various pathological contexts including cytoskeletal proteinopathy (tau), synaptic vesicle–associated aggregation (α-synuclein), expanded polyQ pathology (mHTT), misfolded enzyme-driven ALS (SOD1) and RNA-binding protein mislocalization toxicity (TDP-43). This diversity captures the major mechanistic routes through which the ubiquitin system interfaces with aggregation. While many other aggregating proteins are known in neurodegeneration, such as Amyloid-β, FUS, prion proteins (PrP), optineurin (OPTN), ataxin-2 and ataxin-3, they lack comparable depth in the characterization of their ubiquitin code. Focusing on the above-mentioned five proteins therefore enables a precise and mechanistic exploration of how ubiquitination shapes protein fate in neurodegeneration.

**A**. schematic showing the various domains of tau protein with ubiquitination sites. The colored circles show different types of ubiquitin signatures. Ubiquitination occurs primarily in the microtubule-binding domain of tau. Ubiquitin associated via K48 and K6-linkages directs the tau protein for proteasomal degradation while K11-linked ubiquitin on tau opposes this function. Acetylation of tau at specific lysine residues in the proline-rich domain negatively affects tau degradation. **B**. Schematic showing the domains of α-synuclein protein with ubiquitination (different color circles) and other post translational modification sites. Ubiquitination sites are primarily in the N-terminal domain of α-synuclein. monoubiquitin and K63-linked ubiquitin directs the α-synuclein for lysosomal degradation and its proteasomal degradation is promoted by K48 ubiquitin linkages. **C**. schematic showing the domain organisation and putative ubiquitination sites on mHTT. The colored circles represent different types of ubiquitination. K48-linked ubiquitin on mHTT directs the modified mHTT for proteasomal degradation. The N-terminal ubiquitination sites can also be modified by SUMOylation and cause the aggregation of mHTT. **D**. schematic showing the ubiquitination sites on mSOD1. The K48-linked ubiquitin leads to the proteasomal degradation of mSOD1. The colored circles show different types of ubiquitination. **E**. schematic of TDP-43 domains and their ubiquitination sites. The colored circles show different types of ubiquitin signatures. The RRM1 domain is the major ubiquitination site for TDP-43. The K48-linked TDP-43 is degraded via the proteasome while the K63-linked ubiquitin chains on TDP-43 promote its lysosomal degradation.

**Table 1:** Ubiquitin modifiers and functional consequences of ubiquitination across aggregating proteins

Aggregating proteins	Major E2 conjugates/ E3 ligases / DUBs	Ub chain types observed	Aggregation stages modified	Cellular compartments	Functional outcomes of ubiquitination
**Tau**	E3 ligases: CHIP	Mono-Ub, K6, K11, K48, K63	Oligomers, PHFs, Neurofibrillary tangles	Neuronal (neurofibrillary tangles)	K48 ubiquitin chains promote degradation, K63 ubiquitin chains support lysosomal degradation, K63 ubiquitin chains drive fibrillation and mislocalization, branched chains regulate turnover of pathological tau
**α-Synuclein**	E3 ligases: SIAH-1, Parkin, CHIP, NEDD4, SCF; DUB: USP8, USP9X	Mono-Ub, (K6/K10/K12/K21/K23 / K31/K34 / K43/K96), Di-Ub, K63, K48	Oligomers [49], Fibrils, Lewy bodies	Neuronal (lewy bodies), glial (cytoplasmic inclusions) [50]	K48 ubiquitin chains facilitate proteasomal degradation of aggregates, K63 ubiquitin chains promote endosomal trafficking and lysosomal clearance
**Mutant huntingtin (mHTT**)	E3 ligases: Ube3A, CHIP, TRAF6, WWP1	M1-linked Ub, K6, K11, K27, K29, K48, K63	Insoluble aggregates	Neuronal (intranuclear inclusion) [51] astrocytes	K48 ubiquitin chains promote proteasomal mHTT turnover, K63 ubiquitin chains promote inclusion formation and autophagy
**SOD1**	E2 conjugates: HIP2 E3 ligases: Parkin, CHIP, Dorfin, NEDL-1	Mono-Ub, K48, K63	Fibrils	Neuronal, astrocytes	K48 ubiquitin chains target misfolded SOD1 to proteasome, K63 ubiquitin chains facilitate aggresome formation and autophagy
**TDP-43**	E3 ligases: Parkin, CUL2/Znf179	K48, K63	Fibrils	Neuronal (cytoplasmic inclusions)	K48 ubiquitin chains help in turnover via UPS, K63 ubiquitin chains degrade pathological inclusions via autophagy

### Tau isoforms in Alzheimer’s disease

Tau is a microtubule-stabilizing protein critical for axonal integrity under physiological conditions. Its initial association with neurological diseases was established by antibody reactivity assay, which demonstrated it to be the core protein of intracellular neurofibrillary tangles seen in AD [52,53]. In the adult brain, tau is encoded by the MAPT gene which produces six forms of tau through the alternative splicing of exons 2, 3 and 10. Exons 2 and 3 generate 0N, 1N or 2N N-terminal inserts that regulate membrane interactions and axonal transport. Exon 10 inclusion yields either three (3R) or four (4R) microtubule-binding repeats, with 4R tau showing higher microtubule affinity and greater stabilizing capacity than 3R tau. These structural differences influence how each isoform interacts with the cytoskeleton and its propensity to misfolding and aggregation. While the 3R and 4R tau isoforms together are responsible for Alzheimer’s disease, the 3R tau isoform is more dominant in Pick’s disease, and the 4R isoform leads to Progressive Supranuclear Palsy and corticobasal degeneration [54].

### Ubiquitin signatures on tau

Ubiquitination is one of the numerous changes associated with disease-related properties of tau, although whether these changes are causative for the disease or simply consequences of its altered state is not well understood. Early studies identified ubiquitin as a constituent of SDS-insoluble paired helical filaments (PHF) of tau [55]. Later, Morishima et al. demonstrated that ubiquitin in PHFs of tau found in the brains of AD patients is localised to its microtubule binding region both as monoubiquitin and as Lys48-linked multiubiquitin. Lys254, 257, 311 and 317 in the microtubule binding region (MBD) of tau in PHFs were shown to be modified by ubiquitin. Interestingly, the proteasomal clearance of ubiquitinated PHFs was impaired due to the large size and tight packaging of these aggregated forms, which prevented proteases from accessing this conformation of tau [56]. Ubiquitination of soluble PHFs at other lysine residues in the MBD, such as Lys254 and Lys353, with K6, K11 and K48 polyubiquitin linkages, suggests that residue-specific and code-dependent conformational dynamics of tau underlie disease pathology [57]. A possible explanation for the accumulation of these ubiquitinated species can be the contradictory role of ubiquitin linkages at Lys6 and Lys48, with K6 shown to have reduced ability to stimulate ATP-dependent protein degradation [58]. Figure 1A maps the different lysine residues on tau which are subjected to mono or poly ubiquitination. Ubiquitination at Lys353 and Lys369 of soluble tau has been specifically linked to deposits of the 4R tau protein isoforms [59]. Quantitative proteomics of tau from different stages of AD patients not only supported these findings but also demonstrated ubiquitination as an early disease event, appearing even at the symptomatic stages [60]. As tau pathology increases, proteins involved in ubiquitination and protein quality control become progressively dysregulated, indicating mounting proteostatic stress [61]. Tau aggregates are also modified by K63 ubiquitin linkages for lysosomal degradation. Notably, the constitutive inactivation of p62, a lysosomal adaptor protein, in AD mice results in the hyperphosphorylation, conformational changes and aggregation of K63-linked ubiquitinated tau [62,63]. K63 polyubiquitin linkages have been recently identified to be associated with soluble tau oligomers that are secreted into extracellular spaces for pathological propagation [64].

Ubiquitination of tau exerts distinct, site-specific effects that can be explained in the context of its structure [65,66]. Modifications within the microtubule-binding repeats can sterically block β-sheet formation and suppress fibrillization of tau. It can also reduce microtubule affinity, thereby increasing the pool of soluble tau that is prone to nucleation of tau or other aggregating proteins. Hence, this ubiquitination has dual impact and is dependent on the cellular environment. Ubiquitination in the proline-rich region diminishes microtubule binding and destabilizes long-range intramolecular contacts, thereby exposing aggregation-prone sequences. C-terminal ubiquitination supports aggregation by disrupting tau’s solubilizing tail and promoting filament growth by enhancing access to core β-forming motifs. These region-dependent effects demonstrate how ubiquitination finely tunes tau’s conformational ensemble, solubility and propensity for aggregation.

### Ubiquitin signature modifiers of tau

**Figure 2:**
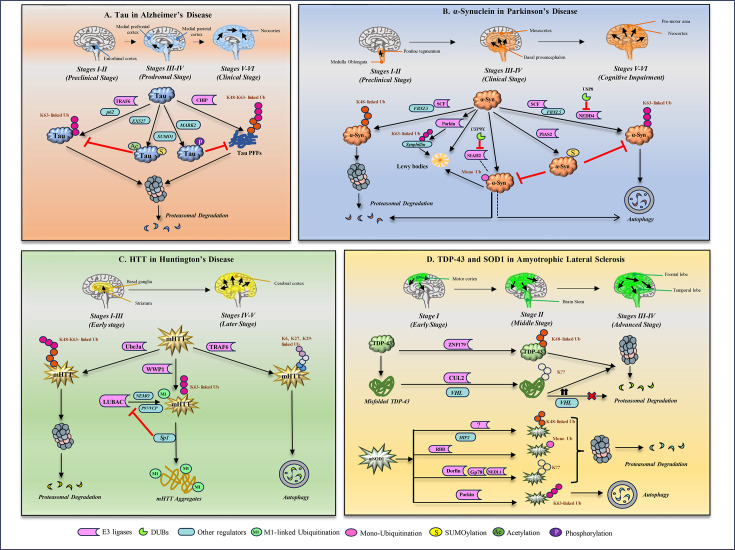
**Disease progression and the fate of ubiquitin modifications on aggregating proteins involved in neurodegeneration**.

CHIP (C-terminus of Hsc70 interacting protein), an E3 ubiquitin ligase, ubiquitinates tau PFF with both K48 and K63 linkages at specific sites *in vitro* [67]. *In vivo* inhibition of the proteasome demonstrates the accumulation of multimeric ubiquitinated tau forms and suggests that CHIP degrades soluble tau in a proteasome-dependent manner [68]. The intrinsically disordered nature of tau influences its accessibility to E3 ligases, but other post-translational modifications also affect E3 ligase interactions with tau. Interestingly, phosphorylation of tau at pS416 by MARK2 kinase reduces its interaction with CHIP, suggesting the role of alternative modifications on ubiquitination of tau [69]. Figure 2A illustrates how ubiquitin modifiers and modifications dynamically shape the turnover of tau. SUMOylation competes with ubiquitination on shared lysine. It also facilitates the hyperphosphorylation and accumulation of aggregated tau, thereby compromising its ubiquitin-mediated degradation. Site-specific mutagenesis of tau SUMOylation (K340R) or inhibition of the same by ginkgolic acid mitigates the effect of SUMO1 and reduces the tau aggregation [70]. Similarly, acetylation blocks ubiquitination at critical positions, weakens microtubule binding and increases the cytosolic pool of aggregation-prone tau. Enhanced acetylation of tau also weakens ubiquitin-dependent degradation of tau. Acetylation at lysine residues (K163, K174 and K180) by an acetyltransferase p300 reduces ubiquitination and clearance of tau. Similarly, inhibition of lysine deacetylase SIRT1 by EX527 enhances tau acetylation and phosphorylation, hence decreasing polyubiquitination and tau degradation [71]. Collectively, these findings highlight the complex and subtle role of ubiquitin signatures in regulating tau turnover, aggregation and toxicity in disease pathology.

The upper panel shows the human brain regions affected at different stages of specific protein aggregation disease, and the lower panel shows the effects of ubiquitin modifications on the specific aggregating proteins. **A**. Tau undergoes K63-linked ubiquitination by TRAF6 E3 ligase, leading to proteasomal degradation via the p62 shuttle. Tau pre-formed fibrils (PFFs) get ubiquitinated through K48/K63 linkages by CHIP E3 ligase and preferentially degraded by the proteasome. Other PTMs like sumoylation, phosphorylation, and acetylation facilitated by SUMO1, MARK2, and EX527 affect ubiquitinated tau degradation negatively. **B**. The SCF E3 ligase complex ubiquitinates α-synuclein via K48 and K63 linkages and catalyzes its proteasomal and autosomal degradation via FBXL5, respectively. SIAH2 monoubiquitinated α-synuclein gets degraded by the proteasome and also leads to toxic inclusions under pathological conditions. When the proteasome is impaired, USP9X inhibits SIAH2 and mediates autophagic clearance of α-synuclein. Parkin ubiquitinates synphillin via K63-linked ubiquitination, resulting in the formation of Lewy bodies. NEDD4 ubiquitinates α-synuclein, via K63 linkages, contributing to its autophagic clearance. Contrarily, the SUMO E3 ligase PIAS2 sumoylates α-synuclein and further inhibits SIAH2 and NEDD4-mediated α-synuclein ubiquitination and degradation. **C**. Ube3a E3 ligase ubiquitinates mHTT via K48-K63 linkages, accelerating its proteasomal degradation, whereas TRAF6 induces K6, K27 and K29 linked mHTT ubiquitination, leading to degradation by autophagy. However, WWP1 promotes the accumulation of mHTT aggregates via its K63-linked ubiquitination. LUBAC associates K63-linked ubiquitin chains at M1 residue of mHTT with the help of proteins like NEMO, p97 and VCP. In diseased conditions, dysfunctional sp1 inhibits LUBAC expression, thus causing aggregation of mHTT. **D**. TDP-43 and SOD1 both contribute to ALS. E3 ligase ZNF179 ubiquitinates TDP-43 via K48 linkages, promoting its proteasomal degradation. Misfolded forms of TDP-43 get recognised by CUL2 E3 ligase along with VHL protein, leading to its degradation by the proteasome. Excess VHL levels, in turn, promote TDP-43 aggregation, thus alleviating its degradation. Mutant SOD1 undergoes mono and K63-linked ubiquitination via RBR and Parkin E3 ligases, facilitating proteasomal and autophagic degradation, respectively. HIP2, an E2 conjugating enzyme, along with an unknown E3 ligase, leads to K48-linked mutant SOD1 ubiquitination, thus promoting proteasomal degradation. Dorfin, Gp78 and NEDL1 are three other E3 ligases also known to polyubiquitinate SOD1 through an unknown mechanism, but lead to SOD1 degradation by the proteasome.

### ⍺-Synuclein in Parkinson’s disease

⍺-Synuclein is a small intrinsically disordered protein that is widely expressed in neurons of the central and peripheral nervous system, blood cells and various other tissues [72]. Its disordered structure partially folds upon binding to negatively charged, highly curved membranes such as synaptic vesicles. Membrane association induces the N-terminal region to adopt an amphipathic α-helical structure, while the hydrophobic NAC domain (which drives aggregation) and the acidic C-terminal tail remain largely disordered. While its conformational flexibility enables physiological roles in vesicle trafficking, it also predisposes the NAC region to misfolding and fibril formation in Parkinson’s disease [73]. It plays a critical role in regulating exocytosis by interacting with synaptic vesicle proteins and in endocytosis via a functional interaction with clathrin during synaptic transmission [74,75]. Monomeric ⍺-synuclein co-exists with its folded tetrameric form under physiological conditions, and an imbalance in the ratio between these two conformers contributes to pathological aggregation [75]. Misfolded ⍺-synuclein accumulation as Lewy bodies (LBs) and Lewy neurites (LN) is considered the pathological hallmark in PD [76]. Partial unfolding of the N-terminus and subsequent exposure of the non-amyloid component (NAC) region promotes the oligomerization of synuclein which represents its most toxic form. The oligomers can further assemble into protofibrils and mature into a highly ordered fibrillar structure characterized by a cross beta-sheet conformation [77]. The distribution and neurotoxicity of pathological α-synuclein, with cell-to-cell transmission of the aggregates through interconnected brain regions, are indicative of the severity and related clinical symptoms of PD [78]. Although the motor symptoms associated with PD are largely due to the dopaminergic loss at the substantia nigra, PD brain patient studies revealed the spread of LB pathology at the extranigral sites and throughout the brain [79]. Braak and colleagues examined over 168 PD brain samples to characterize the disease severity as a staging system, with affected areas initiating from the olfactory bulb to the brain stem, limbic areas and neocortical regions at later stages [79].

### Ubiquitin signatures on ⍺-synuclein

⍺-Synuclein aggregates accumulate diverse ubiquitin linkages which determine whether the aggregates are routed towards proteasomal or autophagy-lysosomal clearance pathways [73]. Some early studies demonstrate that LBs have been consistently stained positively with anti-ubiquitin antibodies, with ubiquitin localizing to the peripheral rim of the inclusions [80,81]. However, a subsequent study revealed that α-synuclein and ubiquitin are either evenly distributed across the inclusion bodies in a homogeneous pattern, or ubiquitin resides in the central domain of LBs with α-synuclein in the periphery in a concentrated pattern [82]. Immunoblot analysis has demonstrated that most of the insoluble ⍺-synuclein species in LBs are either mono- or diubiquitinated, while polyubiquitination occurs less frequently [83,84]. The site-specific ubiquitination on α-synuclein (Figure 1B) provides essential details to understand the fate of the protein [85]. Mass spectrometry analysis identified lysine residues K12, K21 and K23 as ubiquitination sites on α-synuclein, with these modifications predominantly occurring on the phosphorylated form of the protein deposited in LBs [86]. *In vitro* and *in vivo* studies revealed residues K6, K10 and K12 as major ubiquitination sites on filamentous ⍺-synuclein, located at the N-terminal flanking region accessible to ligases, whereas soluble ⍺-synuclein was ubiquitinated at K21, K23, K32 and K34, which are close to the core region of the protein [87]. An *in vitro* assay where modified ⍺-synuclein forms were incubated with 26S proteasome demonstrated that the degradation of monoubiquitinated ⍺-synuclein at residues K6, K12, K31 and K34 occurs at a faster rate compared with unmodified ⍺-synuclein [88]. Notably, a disulphide-directed ubiquitination strategy identified three modification sites (K6, K23 and K96) on α-synuclein that are responsible for preventing the formation of synuclein fibrils [89]. While ubiquitination of α-synuclein at K10 and K23 leads to the formation of filamentous aggregates, a moderate inhibition of fibrillation is observed upon ubiquitination at K6, K12 and K21. Ubiquitination sites K32, K34, K43 and K96 are putative for complete inhibition of fibril formation. Further, a detailed mechanistic study revealed that ⍺-synuclein ubiquitination at K12 significantly inhibits fibrillation and mediates its degradation [90]. Some of this site-specific ubiquitination of α-synuclein can be best explained by how its conformational state reshapes lysine accessibility and the structural context of the NAC aggregation core. In the soluble monomer, residues near the NAC boundary (K21, K23, K32, K34) remain exposed and are readily modified, whereas in filamentous α-synuclein, the core becomes buried, leaving only the N-terminal flanking region (K6, K10, K12) accessible to E3 ligases. Modification of the N-terminal lysine further directs α-synuclein for proteasomal degradation. A study using the Drosophila PD model investigated the role of ubiquitin chain linkages on α-synuclein-induced toxicity. Co-expression of Ub attenuates α-synuclein-induced neurodegeneration, primarily through the formation of K48 linked chains that promote the proteasomal degradation of the aggregates [91]. The semisynthetic conjugation of K48 linked di- and tetra-ubiquitin chains at lysine 12 serves as a signal for proteasomal degradation of α-synuclein [92]. A recent study using mass spectrometry-based analysis suggests that the K63 linked ubiquitination of α-synuclein at K45, K58 or K60 residues is crucial for its endosomal trafficking and subsequent lysosomal degradation [93], elucidating the intricate role of different ubiquitin chain linkages in determining the fate of synuclein. Figure 2B depicts the ubiquitin-dependent regulation of α-synuclein fate.

### Ubiquitin signature modifiers of ⍺-synuclein

Several E3 ligases or E3 ligase complexes, including seven in absentia homolog (SIAH-1), Parkin, CHIP, neuronal precursor cell-expressed, developmentally down-regulated gene 4 (Nedd4), the SKP1-CUL1-F box protein complex and E6-associated protein (E6-AP), play a crucial role in regulating ubiquitin modifications on α-synuclein (Figure 2B). Depending on the type of ubiquitination, these ligases can either help to protect the cells by preventing toxic α-synuclein buildup or, conversely, contribute to disease pathology by promoting aggregation [73,85,94].

Mass spectrometric analysis demonstrates that endogenous SIAH-1 monoubiquitinates ⍺-synuclein at several lysine residues (K10, K12, K21, K23, K34, K43 and K96) [95]. Furthermore, *in vitro* and *in vivo* studies in dopaminergic cells demonstrated that SIAH-1 mediated monoubiquitination of α-synuclein promotes the formation of toxic inclusions [86,95]. Knockdown of SIAH-1 in SH-SY5Y cells reduced synuclein monoubiquitination and aggregation, suggesting the activation of proteasome. Paradoxically, *in vitro* studies also demonstrate that the 26S proteasome can efficiently degrade the monoubiquitinated α-synuclein. Monoubiquitination of α-synuclein at its N-terminal domain consistently promotes its degradation. In contrast, monoubiquitination of centrally located lysine residues shows less or no protein turnover, again suggesting that there are multiple conformational states of α-synuclein which are precisely reshaping the lysine accessibility to the E3 ligases [88]. Under the proteasomal impairment, USP9X deubiquitinase was found to inhibit SIAH-1-mediated synuclein monoubiquitination, thus facilitating its autophagic degradation [96,97]. These findings highlight the complex role of SIAH-1 in α-synuclein turnover, underscoring the need for further investigation to clarify its dual impact on aggregation and clearance.

The HECT E3 ligase Nedd4 also plays a pivotal role in regulating α-synuclein levels by ubiquitinating it at specific lysine residues (K21, K45, K58, K60 and K96) via the K63 polyubiquitin chain, which promotes α-synuclein degradation by the endo-lysosomal pathway. On the contrary, the E3 SUMO ligase PIAS2 promotes α-synuclein SUMOylation and inhibits its ubiquitination by SIAH1 and NEDD4 E3 ligases. This, in turn, leads to the accumulation and α-synuclein inclusion formation, suggesting a reciprocal interplay between ubiquitination and SUMOylation [98]. Overexpression of ubiquitin-specific protease 8 (USP8), a deubiquitinase, in HEK293 cells leads to the selective removal of K63 linked ubiquitin chains from α-synuclein. In a Drosophila PD model, knockdown of USP8 resulted in a reduction of α-synuclein aggregates in an autophagy-dependent manner via conjugation of the K63 linked Ub chain generated by Nedd4 [99]. This evidence collectively highlights the predominant role of Nedd-4 in mitigating PD-associated pathology.

The SCF (SKP1-CUL1-F box) protein complex, a member of the Cullin-RING E3 ligase family, has also been identified as a critical regulator of α-synuclein ubiquitination. Silencing of SKP1 and Cul1 in neuronal cells led to increased accumulation of internalized ⍺ synuclein. Inhibitor studies using MG132 and Baf-A confirmed that SCF ubiquitinates ⍺-synuclein at all N-terminal lysine residues (K10, K45, K58 and K60) via K48-K63-linked chains and targets it for degradation through both proteasomal and lysosomal pathways. Moreover, *in vivo* studies in mice revealed that SCF inhibited the seeding and propagation of extracellular α-synuclein, thereby counteracting LB-like pathology through FBXL5-catalyzed-α-synuclein ubiquitination [100].

The involvement of multiple E3 ligases and residue-specific ubiquitination effects suggests that ubiquitination alters α-synuclein behaviour through site-specific electrostatic shifts, steric occlusion and disruption of lipid-binding helices. N-terminal ubiquitination of α-synuclein weakens membrane binding and increases its aggregation. Ubiquitination near the NAC region may either block its interactions with the C-terminal end exposing the NAC to NEDD4 dependent ubiquitination for degradation or stabilize oligomers by encouraging inter-oligomer contacts. Ubiquitination at the C-terminus might hinder the conformational flexibility of the protein tail. This structural rigidity can favour fibril-compatible conformations, allowing NAC–NAC interactions to occur more efficiently and enabling more stable β-sheet stacking. Hence, ubiquitin signatures dictate the fate of α-synuclein by altering its overall charge, solubility and reorganize long-range intramolecular contacts in α-synuclein’s ensemble.

### mHTT in Huntington’s disease

HD is a fatal autosomal dominant neurodegenerative disease caused by an expanded CAG repeat in the huntingtin (HTT) gene, resulting in a mutant HTT (mHTT) protein with an abnormally long Polyglutamine (PolyQ) expansion, which is prone to form β-sheet rich fibrils. The PolyQ tract lies immediately downstream of an N-terminal membrane binding domain. Following the PolyQ is a polyproline and C-terminal HEAT domain which contribute to the intra and inter domain interactions respectively. As HD progresses, there is marked degeneration of striatal neurons, resulting in significant structural and functional impairment of the striatum within the basal ganglia [101]. In addition, it is characterized by the progressive loss of grey matter specifically in the caudate and putamen during disease onset and subsequently extends to the cerebral cortex. Accumulation of ubiquitin-enriched intranuclear inclusion bodies containing mHTT is one of the pathological hallmarks of HD [55,102].

### Ubiquitin signatures on mHTT

Only nine ubiquitination sites have been identified on the HTT till date (Figure 1C), with lysine residues K6, K9, K631 and K2097 being associated with its mutant form. The deletion of these lysine residues results in an increased accumulation of insoluble mHTT fractions, thereby exacerbating neuronal toxicity and contributing to cell death. Notably, the N-terminal K6 and K9 residues are also subject to SUMOylation (Figure 1C). Despite these findings, the precise molecular mechanism and the functional interplay between ubiquitination and SUMOylation remain unclear [103,104]. Mass spectrometry-based analysis has revealed the early accumulation of K48 linked polyubiquitin chains in both mouse and human models of HD. Additionally, K48, K63 and K11 linkages are also elevated in HD model and patient samples, suggesting a broader disruption of ubiquitin signalling that affects not only proteasomal degradation but also other cellular processes such as cell signalling, cell cycle regulation and DNA repair [102]. Figure 2C summarizes how different ubiquitin linkages govern mutant huntingtin processing in Huntington’s disease. Interestingly, mHTT exhibits region and site-specific consequences of ubiquitination that reflect its structural organization. Ubiquitination of the region flanking the PolyQ tract can block β-sheet assembly while simultaneously altering turnover. In the C-terminal HEAT-repeat domain, ubiquitination destabilizes long-range conformational restraints, exposing the aggregation-prone N-terminal and PolyQ regions and promoting fibril formation. These effects mirror the structural logic seen in tau, underscoring that ubiquitination can either suppress or accelerate aggregation depending on the local context of the modification.

### Ubiquitin signature modifiers of mHTT

The cellular fate of mutant huntingtin (mHTT) is critically influenced by the nature of ubiquitin chains attached to it, which may further depend on its structural flexibility and PolyQ expansions. In a mutagenesis study conducted by Bhatt et al., the authors demonstrated well that mutation of K63 (K63R) caused a marked reduction in mHTT ubiquitination, indicating that K63-linked chains are the predominant modification, whereas K48R had a lesser effect. They also demonstrated that the E3 ligase Ube3a selectively interacts with mHTT, promoting the degradation of specific fragments while reducing aggregation, thereby highlighting its role in regulating mHTT stability in a linkage- and fragment-dependent manner [105]. CHIP also exhibits the ability to polyubiquitinate mHTT in a cell-type-specific manner. In HD knock-in astrocytes, CHIP facilitates K48 linked ubiquitination of mHTT, thereby promoting its proteasomal degradation [106]. Another report demonstrates that TRAF6 E3 ligase ubiquitinates both wildtype and mHTT via atypical linkages including K6, K27 and K29. TRAF6 expression is also elevated in postmortem HD brains with the accumulation of insoluble protein fractions, suggesting its pathological role in mHTT aggregation [107]. In contrast, the E3 ubiquitin ligase WWP1 promotes the accumulation and toxicity of mHTT by forming atypical K63 linked ubiquitin chains, highlighting its pro-pathogenic function in HD [108].

LUBAC (Linear Ubiquitin Chain Assembly Complex) is the only known E3 ligase that is known for the formation of M1-linked linear ubiquitin chains. LUBAC consists of three core proteins, namely HOIP (HOIL-1-interacting protein), HOIL-1L (Haem-Oxidized IRP2 Ubiquitin Ligase 1) and SHARPIN (SHANK-Associated RH Domain Interacting Protein), which are regulated by transcription factor Sp1. The linear ubiquitination plays a crucial role in managing misfolded proteins, such as mHTT, in HD. LUBAC adds M1-linked ubiquitin to the mHTT protein that is already modified by the K63 linked ubiquitin chain. Recruitment of LUBAC requires p97/VCP to coat the mHTT aggregates with a linear Ub chain and form a feedback loop facilitating higher ubiquitination that aids in its proteasomal degradation. In HD models, Sp1 positively regulates LUBAC and reduced activity of Sp1 results in reduced expression of HOIP, HOIL-1L and SHARPIN, which impairs M1 ubiquitination, leading to the accumulation of misfolded proteins causing toxicity [109,110]. Together, these findings highlight the intricate and pathway-specific roles of different ubiquitin linkages in controlling the fate of misfolded and aggregating HTT, providing valuable insights into therapeutic strategies to combat this aggregating protein.

### SOD1 and TDP-43 in ALS

Superoxide dismutase 1 (SOD1) is a small, highly conserved enzyme that converts superoxide radicals into less reactive species. In ALS, numerous inherited mutations destabilize SOD1, causing it to misfold and assemble into intracellular aggregates. These toxic forms of SOD1 disrupt mitochondrial function, strain the protein-quality control machinery and ultimately accumulate as ubiquitin-positive inclusions in motor neurons, making SOD1 misfolding a defining feature of many familial ALS cases [111].

TDP-43, by contrast, is strongly associated with sporadic ALS, which accounts for the vast majority of ALS diagnosis [112]. Normally a nuclear RNA-binding protein involved in splicing, RNA stability and transport, TDP-43 becomes mislocalized to the cytoplasm in ALS, where it undergoes fragmentation and post-translational modifications before forming insoluble aggregates. The combined loss of its nuclear roles and the toxicity of its cytoplasmic inclusions leads to widespread disruption of RNA metabolism in affected neurons.

Although SOD1 mutations drive only a subset of ALS, and TDP-43 pathology is nearly universal in sporadic disease, both proteins follow a common pathogenic path: misfolding, abnormal ubiquitination and failure of degradation systems. This convergence highlights how diverse triggers converge to impair proteostasis, driving motor neuron degeneration.

### Ubiquitin signatures on SOD1

Several mutations in the *SOD1* gene result in non-native folding and aggregation of SOD1, which in turn drive motor neuron degeneration, causing familial Amyotrophic Lateral Sclerosis (fALS). While in the earlier stages, SOD1 aggregates are more prevalent in specific regions of the brain, such as the temporal cortex, later stages affect other regions, including the brainstem and the spinal cord. Mutant SOD1 (A4VSOD1) aggregates have been demonstrated to sequester ubiquitin early during aggregate formation, leading to a depletion of the free ubiquitin pool and marked dysfunction of the ubiquitin–proteasome system (UPS) in cells [111]. Ubiquitin accumulation and mutant SOD1 inclusions in the astrocytes marked the initial clinical onset, characterized by motor neuron degeneration. With disease progression, mutant SOD1 toxicity was exacerbated, mostly due to proteasome impairment [113]. Mutant SOD1 is known to be mono- or polyubiquitinated, mainly with K48 and K63 ubiquitin chains, leading to proteasomal or lysosomal degradation (Figure 1D). Only a single potential lysine site (K136) has been identified to date as being involved in the ubiquitination of SOD1, specifically in K48 ubiquitin chain conjugation. However, contributions of other lysine sites for SOD1 ubiquitination remain to be further elucidated [114].

### Ubiquitin signature modifiers of SOD1

SOD1 expression and function is regulated by various components of the UPS pathway, with multiple E3 ubiquitin ligases involved in its degradation (Figure 2D). Dorfin was the first identified RING-type E3 ligase that ubiquitinates mutant SOD1 at K137 but not WT SOD1 and thus reduces mutant SOD1 inclusions, thereby targeting them for proteasomal degradation [115]. To enhance the Dorfin E3 activity towards mutant SOD1, chimeric CHIP-Dorfin proteins are developed and shown to rapidly degrade mutant SOD1 compared with WT SOD1 *in vitro* and also rescue neuronal cells from SOD1 cytotoxicity [116]. NEDL-1, homolog of E6-AP, in concert with the endoplasmic reticulum translocon-associated protein TRAP-δ, ubiquitinates mutant SOD1 aggregates, thereby decreasing the mutant function. However, the interaction of NEDL-1 with mutant SOD1 affects its physiological function, leading to neuronal apoptosis and motor neuron death [117]. This might be a contributing factor in the pathogenesis of fALS, and hence, further studies may provide insights into the mechanism behind the formation of these complexes and their consequences. Proteomic characterization of detergent insoluble fraction of spinal cord fluid from G93A SOD1 mice identified K163 as a potential site for monoubiquitination of oligomers [114]. Mutant SOD1 also localizes to mitochondria and triggers mitochondrial dysfunction, inducing early onset and progression of ALS. It is reported that when the proteasome is impaired, Parkin mediates K63 linked ubiquitination of mutant SOD1, serving as a cargo signal for mutant SOD1 aggresome formation and clearance by autophagy pathway [118]. Huntingtin-interacting protein 2 (HIP2), an E2 ubiquitin-conjugating enzyme, also plays a role in regulating mutant SOD1 aggregates. It has been demonstrated that in an ALS disease model, the levels of HIP2 are elevated by mutant SOD1, and shRNA-mediated knockout of HIP2 results in a significant reduction in the ubiquitination of mutant SOD1. Furthermore, a mutation in the K48 ubiquitin leads to a decrease in the ubiquitination of mutant SOD1, indicating that it is polyubiquitinated through a K48 ubiquitin linkage. Inhibition of the proteasome by MG132 promotes the accumulation of mutant SOD1, suggesting that its degradation occurs via UPS [119]. Considering that both UPS and autophagy pathways actively clear SOD1 aggregates, this suggests that overwhelming of one degradation route by other cellular aggregates can reduce or enhance the aggregate phenotypes of SOD1.

### Ubiquitin signatures on TDP-43

Mislocalization of TDP-43 inclusions in the cytoplasm of neurons is a major hallmark of ALS, leading to motor neuron degeneration [120]. TDP-43 pathology initiates at the prefrontal neocortex (Stage I) and progresses to other regions such as the hippocampus and thalamus (Stage II-III), with the highest burden of TDP-43 deposits in the occipital neocortex at the advanced stage (Stage IV) [121]. Under physiological conditions, TD-43 localizes to the nucleus, but it is present as ubiquitin-modified cytoplasmic inclusions in patient brains affected with frontotemporal lobar degeneration and ALS [11]. Numerous ubiquitination sites have been reported for TDP-43, including K79, K84, K95, K102, K114, K121, K140, K145, K160, K176, K181 and K263 (Figure 1E). TDP-43 undergoes both K48 and K63 linked polyubiquitination, resulting in proteasomal and autophagolysosomal degradation, respectively [122,123].

### Ubiquitin signature modifiers of TDP-43

Studies in transgenic A315T mutant TDP-43 (TDP-43-Tg) mice and gene transfer rat models demonstrated that Parkin ubiquitinates TDP-43 (with both K48 and K63 chains) and co-immunoprecipitates with HDAC6, suggesting that Parkin-HDAC6 complex facilitates nuclear export and cytosolic sequestration of mutant TDP-43 potentially contributing to its proteinopathy [124]. But how Parkin regulates cytosolic accumulation of ubiquitinated TDP-43 and either promotes its clearance through degradation is still unclear. The cullin (CUL2) E3 complex, along with von Hippel-Lindau protein (VHL), a substrate-binding component of the complex, recognizes and ubiquitinates misfolded forms of TDP-43. While CUL2/VHL promotes proteasomal degradation of fragmented forms of TDP-43, strikingly excessive VHL leads to the stabilization of aggregate formation at the juxtanuclear protein quality control centre, indicating that an imbalance of CUL2 and VHL may contribute to ALS pathogenesis via oligodendrocyte dysfunction [125]. The critical role of a novel E3 ligase Znf179 has also emerged in TDP-43-mediated ALS neuropathy. Immunoprecipitation and mass spectrometry analysis revealed that Znf179 mediates TDP-43 polyubiquitination and enhances its degradation by the proteasome, via K48 linkages, whereas knockout of Znf179 in mouse brain leads to excessive insoluble TDP-43 in midbrain, cortex and hippocampus regions. This directs the autophagic clearance of TDP-43 insoluble aggregates via Znf179-mediated K63 ubiquitination [126]. Thus, Znf179 functions as a solubility-dependent ubiquitin-topology switch that protects neurons from TDP-43 accumulation. Additionally, mutant TDP-43 and FUS, the other aggregating proteins associated with ALS, disrupt the proteostatic pathways. Elevated levels of K48- and K63-ubiquitinated mutant TDP-43 and SOD1 have been observed, suggesting a possible role for the UPS and autophagy in degrading the aggregates, potentially in a linkage-specific manner [127]. Figure 2D illustrates the ubiquitination pathways regulating the stability of TDP-43, highlighting how specific E3 ligases and linkages influence their degradation in ALS.

### Pro-pathogenic roles of ubiquitination

Examining the various ubiquitin modifications on aggregating proteins in neurodegeneration reveals that ubiquitination plays a crucial and causative role in the pathogenesis of tau, α-synuclein, mutant huntingtin (mHTT), SOD1 and TDP-43 aggregation. For these proteins, specific lysine ubiquitination events directly modulate conformational states, oligomerization kinetics or intercellular propagation, demonstrating that ubiquitination is not simply a passive marker of impaired protein clearance. For example, K48-linked tau ubiquitination directs it for proteasomal degradation [56], while K63 ubiquitination exerts a dual effect: it targets tau for lysosomal degradation [62] and simultaneously promotes the prion-like spread of toxic tau oligomers [64]. Similarly, α-synuclein ubiquitination at K6, K10 and K12 is known to stabilize soluble oligomers and increase seeding efficiency [83], while ubiquitination at K6, K23 and K96 of α-synuclein prevents its fibrillation but might stabilize other on-pathway oligomers [89]. In mHTT, ubiquitination of lysine within the N-terminal domain (including K6 and K9) enhances proteasomal processing and inhibits the accumulation of aggregation-prone fragments, linking the modification pattern to inclusion formation [128]. Detergent-insoluble fractions of disease-linked SOD1 ubiquitination at K163 appear after the aggregate formation [129]. Interestingly, the E3 ligase NEDL-1 adds ubiquitin chains that paradoxically increase SOD1 toxicity by stabilizing intermediate oligomers [117], suggesting that ubiquitin does not simply tag SOD1 for destruction; rather, it defines which misfolded species accumulate in the cell. For TDP-43, ubiquitination by Parkin and an excess of CUL2/VHL enhances pathological inclusion formation even before global proteostasis collapse [124,125]. Studies also show that aggregation of a single misfolded protein can destabilize the broader proteostasis network by sequestering and functionally quenching key protein quality control regulators. In *C. elegans,* PolyQ-expanded proteins trap essential chaperones and folding factors, triggering misfolding of unrelated metastable proteins [130]. This collapse is driven not only by proteasomal or autophagy overload but also by depletion of the regulatory protein quality control machinery itself. These findings strongly suggest how misfolded and ubiquitinated proteins such as TDP-43, mutant SOD1, α-synuclein or mHTT exacerbate co-pathology by weakening the cellular systems that maintain proteome integrity. Although traditionally viewed as a lysine-directed modification, an emerging body of evidence shows that cysteine ubiquitination via thioester or thioether linkages can also occur and may have unique regulatory consequences for aggregation-prone proteins [27]. Interestingly, cysteine ubiquitination has been observed so far only for Parkin, as well as for ubiquitinating TDP-43 and α-synuclein. Most of the aggregating proteins discussed in this review have cysteine residues, and modification at these residues could, in principle, influence their aggregation by altering disulphide bonding, shifting the redox state or perturbing intramolecular stabilizing interactions. However, further studies are needed to establish the pro-aggregating function of non-canonical ubiquitination.

Together, these examples highlight the proactive role of the ubiquitin code in shaping aggregation dynamics, propagation, localization and toxicity. Ubiquitination is therefore not merely a consequence of failed degradation, but a determinant of pathogenic identity, capable of either accelerating or modulating the trajectory of neurodegeneration, depending on the site, chain type and cellular context.

### Ubiquitin-mediated cross-talk among the aggregating proteins

Ubiquitination plays a crucial role in determining how protein aggregates interact with one another during neurodegeneration. For instance, the clearance of α-synuclein becomes markedly impaired in the presence of amyloid-β (Aβ) plaques. When α-synuclein preformed fibrils are introduced into transgenic Alzheimer’s disease mice, Aβ-rich regions show enhanced phosphorylation of α-synuclein and accelerated spread of pathology. These areas also accumulate high levels of K48 and K63 linked ubiquitinated α-synuclein together with p62-positive inclusions, indicating a blockade of both autophagy and proteasomal degradation [131]. Thus, the fate of α-synuclein is dictated not only by its ubiquitin chain architecture but also by the presence of other disease aggregates that reshape the proteostasis environment.

A second layer of co-pathology arises from competition for the shared E3 ubiquitin ligases. Many aggregation-prone proteins depend on the same E3 ligases, particularly CHIP and Parkin (Table 1). When one aggregating protein, such as α-synuclein fibrils or expanded polyQ aggregates, monopolizes CHIP or Parkin, the ubiquitination and turnover of other substrates decline, which might set the stage for secondary aggregation. In ALS, misfolded SOD1 aggregates become heavily polyubiquitinated and sequester p62 and NBR1, reducing availability of these receptors for TDP-43 clearance [132]. Conversely, ubiquitinated TDP-43 inclusions impair proteasomal capacity, or vice versa, which can lead to accumulation of misfolded SOD1 and accelerate its aggregation [133]. Both proteins frequently co-accumulate as ubiquitinated inclusions in patient tissue, reflecting shared clearance bottlenecks that amplify each other’s toxicity [134]. Proteome-wide analyses also support the idea that distinct aggregating species trigger convergent cellular stress responses early in disease. Pichet Binette et al. demonstrated that in the earliest stage of Alzheimer’s pathology, the oxidative-stress–associated proteins such as PARK7, SOD1, SOD2 and GSR are highly dysregulated, indicating that Aβ accumulation alone is sufficient to activate stress pathways that are also implicated in tau, TDP-43 and α-synuclein aggregation [61].

Thus, understanding co-pathology through the lens of ubiquitination reveals why neurodegenerative diseases frequently show overlapping aggregate profiles: not because each protein behaves independently, but because they compete within the same constrained ubiquitin signalling landscape. This interconnectedness helps explain disease severity, regional vulnerability and why clearing a single aggregate species often fails to halt disease progression.

### Conclusions and future directions

Protein aggregation and the ubiquitin system are deeply intertwined in the pathogenesis of neurodegenerative diseases such as Alzheimer’s, Parkinson’s, Huntington’s, ALS and FTD. This review has examined how different types of ubiquitin linkages, site-specific modifications and the 8u6 activities of E3 ligases and deubiquitinases regulate the fate of key aggregation-prone proteins like tau, α-synuclein, mutant huntingtin (mHTT), SOD1 and TDP-43. Ubiquitin chain conformations, including the well-known K48 and K63, and non-canonical linkages like K6, K11, K27, K29 and M1 govern whether a protein is degraded, sequestered or propagated, emphasizing the complex role of ubiquitination beyond simple degradation signals.

Although these proteins are targeted by the proteasome and autophagy pathways, their degradation is frequently inefficient due to the stability of aggregates or the presence of competing post-translational modifications. Notably, certain E3 ligases or ubiquitin chains may facilitate rather than suppress aggregation, reflecting a highly context-dependent and dynamic regulatory system. This dual role demands a broader understanding of ubiquitin signalling as an integrated response to cellular stress rather than a unidirectional degradation pathway. Moreover, lysine residues on these disease proteins are often modified by other post-translational marks such as phosphorylation, acetylation or SUMOylation, which influence their ubiquitination status. These modifications compete or co-operate with ubiquitin, affecting aggregation kinetics, clearance efficiency and intercellular spread. The co-existence of multiple aggregates, as seen in mixed pathologies (e.g., Tau and α-synuclein), may further alter the ubiquitin landscape, indicating the need for integrative studies that address overlapping disease mechanisms.

Moving forward, several research priorities stand out. It is essential to map the full spectrum of ubiquitin chain linkages on disease-associated proteins under both normal and pathological conditions. Recent reports of dominant ubiquitin variants that subtly alter E2/E3 recognition interfaces to redirect ubiquitination flux and remodel substrate fate present these ubiquitin variants as powerful synthetic probes to dissect how aggregates choose between clearance pathways [135,136]. While K48 and K63 linkages are well characterized, atypical chains remain poorly understood. Their identification across disease stages may highlight key regulatory transitions in protein fate. The spatio-temporal dynamics of ubiquitination during disease progression must be elucidated. Most data available today come from late-stage disease tissues, limiting insight into the initial pathogenic triggers. Animal models like *C. elegans*, Drosophila, patient-derived organoids and early-stage human tissue could help identify when protective ubiquitin signalling becomes dysregulated. These models can be used to explore the specificity and regulation of E3 ligases and DUBs. Enzymes such as CHIP, Parkin and NEDD4 have been implicated in modulating neurodegenerative proteins; however, the determinants of their chain preferences, substrate recruitment and activity under stress remain unclear. Defining these parameters will aid in targeting them therapeutically. More complex disease models that replicate the physiological co-pathologies should also be prioritized in future research. These will provide insights into how distinct aggregates affect each other’s ubiquitination and whether shared degradation machinery can be competitively disrupted. Cross-talk between ubiquitination and other post-translational modifications with an emphasis on simultaneous profiling of multiple modifications on the same protein will be critical for understanding their combined impact on aggregation, clearance and toxicity. Ultimately, translating these insights into effective therapeutic strategies is a realistic long-term goal. These may include engineered ubiquitin, E3 ligases or DUB modulators, as well as ubiquitin chain-specific sensors and targeted degradation technologies like PROTACs, which are tailored for aggregation-prone proteins (Table 2). Trim-Away is one such method that uses intracellularly delivered antibodies to recruit the E3 ligase TRIM21, which rapidly ubiquitinates and degrades the bound target protein via the proteasome. This enables fast, gene-independent depletion of endogenous proteins in diverse cell types [146]. The emergence of PROTACs has reshaped targeted degradation strategies for neurodegenerative diseases. The first neurodegeneration-focused PROTAC, TH006, tethered VHL to tau, driving K48-linked tau polyubiquitination and degradation through both autophagy and proteasome-dependent mechanisms; it also reduced Aβ toxicity in AD mouse models [138]. More recently, C004019 induced robust tau degradation through dual pathways and improved cognition when administered subcutaneously in AD mice [139]. These findings highlight how manipulating the ubiquitin code via K48 or K63 linkages offers a powerful avenue for selective clearance of pathogenic proteins in neurodegeneration. A deeper understanding of ubiquitin’s regulatory roles holds promise for developing precise interventions to mitigate protein aggregation and preserve neuronal health.

**Table 2:** Therapeutic strategies and their ubiquitination specific mode of action in neurodegenerative diseases

Therapeutic strategies	Subclasses	Examples	Ubiquitin code-specific mechanisms	Target proteins/Diseases	References
**E3 ligase mediated targeted protein degradation**	Trim-away (TRIM21-mediated degradation)		Hijacks TRIM21 to add K63-linked ubiquitin chains and clear mHTT aggregates.	mHTT- Huntington’s disease	[137]
	PROTACs (protein targeting chimaeras)	TH006	Engages VHL to add K48-linked ubiquitin chains and degrade tau.	Tau-Alzheimer’s disease	[138]
		C004019	Enhances tau ubiquitination for proteasomal clearance.	Tau-Alzheimer’s disease	[139]
	SNIPERs (specific and nongenetic IAP-based protein erasers)	Compounds 1, 2 and 7	Ubiquitinates mHTT aggregates to enhance their degradation.	mHTT- Huntington’s disease	[140,141]
**DUB modulators**		USP14 inhibitor IU1	Inhibits USP14 to accelerate tau and TDP-43 degradation.	Tau-Alzheimer’s disease TDP-43- amyotrophic lateral sclerosis	[142]
		USP13 inhibitor BK50118-C	Blocks USP13 to prevent α-synuclein deubiquitination and promote its degradation.	α-Synuclein- Parkinson’s disease	[143]
		UCHL1 inhibitor LDN-57444	Inhibits UCHL1 to drive autophagic clearance of α-synuclein.	α-Synuclein- Parkinson’s disease	[144]
		USP7 inhibitor HBX41108	Stabilizes NEDD4L to reduce mutant SOD1 toxicity.	SOD1- amyotrophic lateral sclerosis	[145]

**Summary Points**

- Ubiquitination is central to neurodegeneration, regulating the fate of key aggregating proteins such as tau, α-synuclein, mutant huntingtin, TAR DNA-binding protein 43 and superoxide dismutase 1.
- Ubiquitin chain types and linkages form a complex signature that dictates protein clearance or persistence.
- Aberration in the function of E3 ligases or DUBs disrupts proteostasis and accelerates disease progression.
- Targeting the ubiquitin system offers promising therapeutic avenues for neurodegenerative diseases.

## Open Access

This article has been published open access under our Subscribe to Open programme, made possible through the support of our subscribing institutions, learn more here: https://portlandpress.com/pages/open_access_options_and_prices#conditional

## Abbreviations

AD: Alzheimer’s disease
ALS: amyotrophic lateral sclerosis
FTD: frontotemporal dementia
PD: Parkinson’s disease
PROTACs: Proteolysis targeting chimeras
